# The Renin-Angiotensin System Involvement in Cisplatin-Induced Nephrotoxicity: An Overview of Physiological and Pathological Mechanisms—A Systematic Review

**DOI:** 10.1155/2024/1511216

**Published:** 2024-05-18

**Authors:** Aryan Vakilian, Sina Mohammadi, Fatemeh Shokri, Maryam Maleki, Maryam Kheiry, Amin Kheiri

**Affiliations:** ^1^Department of Physiology, Faculty of Medicine, Ilam University of Medical Sciences, Ilam, Iran; ^2^Non-Communicable Diseases Research Center, Ilam University of Medical Sciences, Ilam, Iran; ^3^Department of Endodontics, Faculty of Dentistry, Ilam University of Medical Sciences, Ilam, Iran

## Abstract

Cisplatin (CDDP) is a highly potent chemotherapy drug. But its nephrotoxicity poses a significant limitation to its use. The renin-angiotensin system (RAS) has been proposed to play a role in drug-induced nephrotoxicity. This systematic review (SR) sought to identify the link between CDDP-induced nephrotoxicity and the RAS pathway. In this SR, relevant keywords were employed to explore databases such as PubMed (MEDLINE), Scopus (Elsevier), and Institute for Scientific Information (ISI) Web of Science up to October 2023. Nine studies were selected based on predefined inclusion/exclusion criteria. The findings support the involvement of the RAS in the CDDP-induced nephrotoxicity model, along with the activation of inflammatory mediators, lipid peroxidation, and changes in markers of kidney tissue damage. Furthermore, physiology and pathology of RAS-related interventions in CDDP-induced nephrotoxicity models have involved the factors such as human organic cation transporter 2 (hOCT2), organic anion transporting polypeptides 1B1 (OATP1B1) and 1B3, kallikrein-kinin system, and bradykinin receptors. CDDP-induced nephrotoxicity has been found to be substantially influenced by both classic and nonclassic RAS axes. Angiotensin II exacerbates renal damage induced by CDDP. Conversely, inhibiting the pressor arm of RAS in males mitigates this damage. However, activation of the renal vasodepressor arm of RAS exacerbates CDDP-induced nephrotoxicity in females. These findings underscore gender differences in renal function and response to RAS-related interventions in the presence of CDDP. This SR provides insights into both beneficial and adverse interventions associated with RAS in the CDDP-induced nephrotoxicity, offering valuable considerations for researchers and clinicians.

## 1. Introduction

Cisplatin (cis-diamminedichloroplatinum, CDDP) is a widely employed platinum-based chemotherapy drug for treating various cancers [1]. However, the use of CDDP in chemotherapy carries the inherent risk of causing renal injury [2, 3]. This renal injury is attributed to several mechanisms, including the promotion of cytochrome c release from proximal tubular epithelial cells and the activation of caspase-9 [4]. Furthermore, CDDP leads to the generation of role of tumor necrosis factor alpha (TNF-*α*) in tubular cells, triggering an inflammatory response that ultimately results in tubular cell injury or death [5]. Additionally, CDDP-induced endothelial dysfunction and vasoconstriction directly contribute to the toxicity of tubular epithelial cells, leading to reduced renal blood flow [1].

This renal toxicity poses a significant limitation to the use of CDDP in the treatment of certain types of tumors [2]. The renin-angiotensin system (RAS) has been proposed to play a role in the development of kidney diseases and, consequently, in drug-induced nephrotoxicity [6]. Notably, Arora et al. [7] reported a 27.6% increase in the risk of acute kidney injury (AKI) in patients following cardiac surgery when RAS inhibitors were used. RAS is vital in regulating blood pressure and electrolyte balance [8].

Angiotensin II (Ang II), the primary effector peptide within the RAS, plays a pivotal role in maintaining arterial blood pressure and fluid equilibrium [9]. The RAS is a complex system with both protective and nonprotective components [10]. The key active component in the RAS is Ang II, which has the ability to bind and trigger both Ang II type-1 receptors (AT1R) and Ang II type-2 receptors (AT2R). Meanwhile, Ang-(1–7), another component of RAS primarily derived from Ang II, can bind to and activate the Mas receptor (MasR). Activation of AT1R promotes vasoconstriction, cellular inflammation, proliferation, and fibrosis. In contrast, activation of AT2R and MasR leads to vasodilation and exhibits antiproliferative, antioxidative, anti-inflammatory, and antifibrotic effects [10–12]. Based on the other studies, we posit that RAS and its related therapeutic interventions may play significant physiologic and pathophysiologic roles in the nephrotoxicity. Consequently, the objective of this systematic review (SR) is to review and analyze available findings on the relationship between the “RAS” and “CDDP-induced nephrotoxicity model.”

## 2. Methods: SR Approach

### 2.1. Search Strategy and Selection Criteria

**Figure 1:**
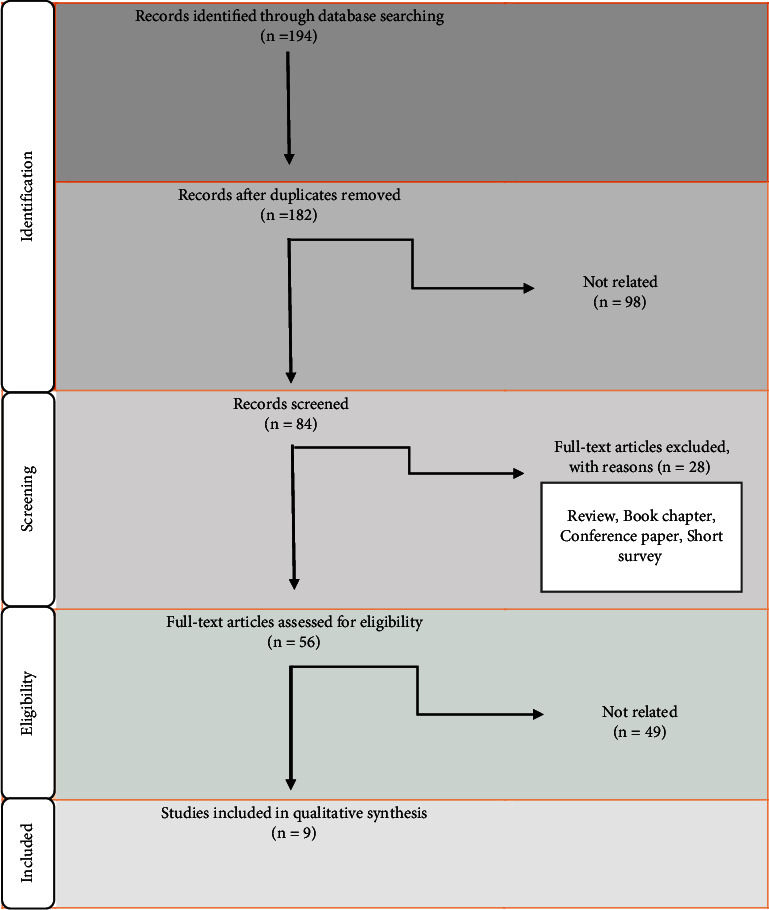
Flow chart of the selection of studies based on PRISMA.

The SR protocol and data extraction forms were designed according to the previous research that our team published [10, 13]. We conducted a systematic search of PubMed (MEDLINE), Scopus (Elsevier), and Institute for Scientific Information (ISI), up to October 2023.  Table 1 shows the search syntax. The systematic literature search identified 194 articles; only 9 fulfilled the inclusion criteria (Figure 1) and finally were subjected to the critical review. M. M conducted the primary data extraction. All articles were examined independently by M. Kh, the second reviewer. The name of the author, research model, publication year, results, dose of drugs, model, receptor(s)/pathways, the authors, animal/human model, injury/debases model, drugs dosage, investigated receptors/pathways, therapeutic interventions/investigations related to RAS in CDDP-induced nephrotoxicity, results, protective/destructive functions, and molecular pathways are all listed, in order of importance.

### 2.2. Inclusion and Exclusion Criteria

Studies met the following inclusion criteria: (1) studies conducted and published as an original paper; (2) studies published in peer-review journals; (3) studies focusing on the connection between the RAS and CDDP-induced nephrotoxicity. Exclusion criteria were review articles, duplicate ones, editorials, brief communications, rules, theses, oral presentations, book chapters, and conference abstracts.

## 3. Result

194 articles were selected from three databanks. Then, 187 articles were disregarded since having nothing to do with the inclusion criteria. Ultimately, after reviewing full texts, 9 articles based on the inclusion/exclusion criteria were selected for this SR. At first, we investigated the nine articles' complete descriptions, a summary of their specifications and principal results based on animal and human models. In the next step, all 9 articles were entirely studied and investigated in depth and categorized according to physiology and pathophysiology of RAS in CDDP-induced nephrotoxicity, which is explained in Tables 2–5.

Table 2 displays the nine articles' complete descriptions and a summary of their characteristics.

Table 3 presents the characteristics of studies indicating the positive therapeutic effects of RAS-related interventions on CDDP-induced nephrotoxicity.

Characteristics of studies that indicate the pathologic effects of RAS-related interventions on CDDP-induced nephrotoxicity are indicated in Table 4.

Characteristics of studies that indicate the effect of CDDP use on the RAS in the nephrotoxicity model are indicated in Table 5.

## 4. Discussion

**Figure 2:**
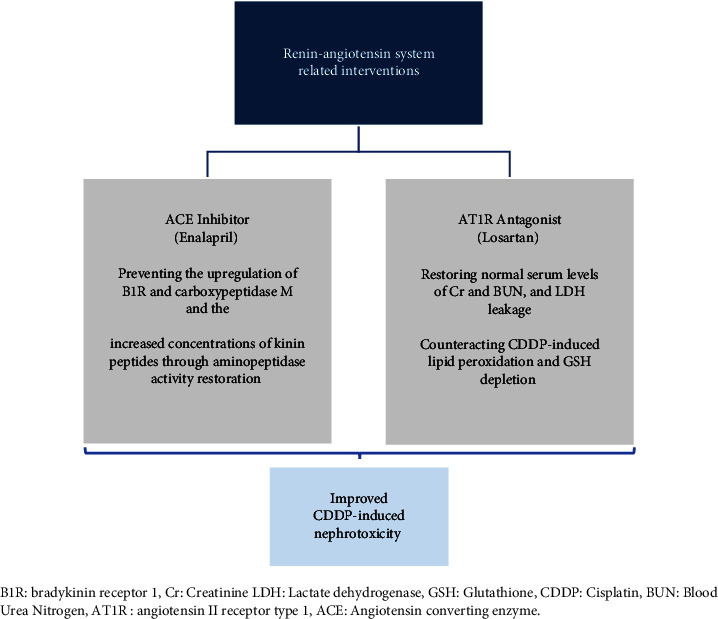
Positive effects of renin-angiotensin system (RAS)-related interventions in cisplatin-induced nephrotoxicity models. B1R: bradykinin receptor 1, Cr: creatinine LDH: lactate dehydrogenase, GSH: glutathione, CDDP: cisplatin, BUN: blood urea nitrogen, AT1R: angiotensin II receptor type 1, ACE: angiotensin-converting enzyme.

**Figure 3:**
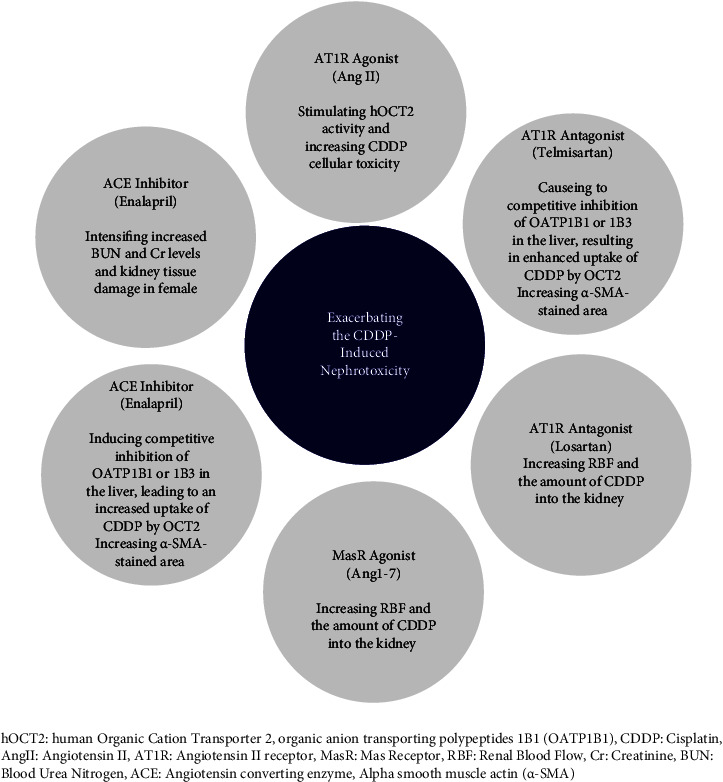
The pathologic effects of the renin-angiotensin system (RAS)-related interventions in cisplatin-induced nephrotoxicity models. hOCT2: human organic cation transporter 2, organic anion transporting polypeptides 1B1 (OATP1B1), CDDP: cisplatin, AngII: angiotensin II, AT1R: angiotensin II receptor, MasR: Mas receptor, RBF: renal blood flow, Cr: creatinine, BUN: blood urea nitrogen, ACE: angiotensin-converting enzyme, alpha smooth muscle actin (*α*-SMA).

The current SR investigates different studies related to the physiology and pathophysiology of RAS in CDDP-induced kidney injury. Characteristics of the studies conducted on the relationship between RAS and CDDP-induced nephrotoxicity are shown in Table 2. These studies have reported that CDDP-induced nephrotoxicity has been found to be substantially influenced by both classic and nonclassic RAS axes. Additionally, some of RAS interventions have protective effects (Figure 2) and some have promoting effects (Figure 3) on CDDP-induced nephrotoxicity. This is covered in more depth in the next paragraphs.

### 4.1. The Effects of RAS-Related Therapeutic Interventions on Cisplatin-Induced Nephrotoxicity

Table 3 shows the characteristics of studies indicating the therapeutic effects of RAS-related interventions on CDDP-induced nephrotoxicity.

Estrela et al. [15] demonstrated that ACE inhibitors confer protection against CDDP-induced nephrotoxicity in mice. This protection is achieved by modulating the expression of kinin B1 receptors (B1R) and enhancing aminopeptidase P (APP) activity. Their findings suggest a potential strategy of employing B1 and B2 receptor antagonists to mitigate renal injury in patients undergoing CDDP treatment. These receptors are activated by kinins, primarily metabolized by ACE. ACE inhibitors, the widely used drugs, reduce kinin degradation [15]. Moreover, Estrela et al. [15] reported that ACE inhibition not only decreases AngII levels but also downregulates the expression of both kinin receptors (B1 and B2R), specially B1R. These show that the protective effects of enalapril (an ACE inhibitor) against CDDP-induced renal injury are attributed to both reduced Ang II production and kallikrein-kinin system (KKS) components' modulation. Enalapril particularly does the latter by reducing carboxypeptidase M (CPM) expression, thereby diminishing kinin B1R activation and its overexpression. This mechanism prevents the elevation of kinin peptide concentrations by enhancing APP activity, exerting an effect on kidney epithelial tubular cells [15].

Saleh et al. [18] found that an angiotensin II receptor blocker (ARB), losartan, significantly prevents lipid peroxidation and glutathione (GSH) reduction induced by CDDP. Thus, in addition to blocking Ang II receptors, its nephroprotective mechanism likely consists of antioxidant properties. So, losartan may have the capability to restore cell defense mechanisms and inhibit lipid peroxidation [18]. However, CDDP uptake in the kidneys did not reveal any significant differences related to losartan; therefore, it is suggested that the nephroprotective effect of losartan may not be via altering cisplatin uptake [18].

These studies declare that inhibition of the pressor arm of RAS (ACE and Ang II receptor) in males reduces the kidney damage caused by CDDP that involves affecting the KKS and antioxidant properties. Studies that emphasize the nephroprotective role of losartan in the CDDP-induced nephrotoxicity have some ideas as prophylactic administration, gender, and drug dose differences. In coadministration models, cisplatin mostly worsens the conditions.

### 4.2. The Pathologic Effects of RAS on CDDP-Induced Nephrotoxicity

Table 4 is the characteristics of studies that indicate the pathologic effects of RAS on CDDP-induced nephrotoxicity.

An organic cation transporter (OCT2), typically abundant in the kidney, plays a significant role in CDDP-induced cellular toxicity and nephrotoxicity [14]. Inhibiting the OCT2 function presents an appealing therapeutic option for mitigating the side effects of platinum-based chemotherapy without compromising their antitumor efficacy [14]. Kantauskaite et al. in their study suggested that Ang II stimulation via enhancing human OCT2 (hOCT2) activity increases CDDP-induced cellular toxicity [14]. Ang II through binding to the AT1R could stimulate hOCT2 function, possibly via protein kinase C (PKC)-dependent and calcium-independent mechanisms [14].

On the other side, Tsuji et al. [1] reported that RAS inhibitors, when administered alongside CDDP, exacerbate CDDP-induced nephrotoxicity in mice. Simultaneous administration of CDDP with an ACE inhibitor (enalapril) or an ARB (telmisartan) worsened kidney damage. Enalapril and telmisartan are known to be absorbed by organic anion transporting polypeptides 1B1 (OATP1B1) and 1B3 in the human liver [1, 21], while CDDP is taken up by OCT2 in the kidney basement membrane, contributing to nephrotoxicity [1]. Additionally, CDDP forms negative complexes with carbonate in the blood [1] and is taken up by the liver similar to enalapril and telmisartan [22]. Consequently, enalapril and telmisartan may competitively inhibit OATP1B1 or 1B3 in the liver, leading to increased CDDP uptake by OCT2 in the kidney basement membrane, thereby exacerbating renal damage and mortality. Furthermore, studies have reported a significant reduction in the renal blood flow (RBF) in rats with CDDP-induced nephrotoxicity [23] which may be exacerbated by concurrent administration of CDDP and enalapril or telmisartan, worsening CDDP-induced renal injury [1]. Also, the development of kidney fibrosis is a mechanism by which the combination of enalapril and telmisartan exacerbates renal damage and mirrors the pathogenesis of renal dysfunction, correlating with serum creatinine and blood urea nitrogen (BUN) levels. Additionally, the concurrent administration of CDDP and enalapril or telmisartan exhibited an increase in the alpha smooth muscle actin (*α*-SMA)-stained area compared to the CDDP group [1] that positively correlates with increased renal fibrosis [1]. Intriguingly, Nematbakhsh et al. reported that not only did enalapril fail to diminish CDDP-induced nephrotoxicity in both female and male rats, but it also exacerbated CDDP-induced nephrotoxicity in females, which can be attributed to gender-dependent differences in RAS and its balancing [17]. It is documented that ACE activity is lower in women compared to men [17]. Therefore, the varying effects of enalapril between genders could be due to RAS response gender-specific differences [17]. Furthermore, Kasaei et al. demonstrated that Ang-(1–7) and losartan exacerbated CDDP-induced nephrotoxicity in female rats. The vasodilatory effects of losartan lead to enhance RBF, increase transport of CDDP to the kidneys, and amplify the undesirable effects of CDDP within the kidneys. This mechanism can also be attributed to Ang-(1–7) [16]. As Ang-(1–7) reduces renal AT1R expression and increases RBF, so it facilitates CDDP transport to the kidney and may perform more kidney damage [16]. However, when MasR was blocked by A779 (MasR antagonist), the effect of Ang-(1–7) on CDDP-induced nephrotoxicity did not significantly change [16].

Nematbakhsh et al. [9] have reported that CDDP raises the vasoconstriction effects of Ang II more in males than females. Possibly, CDDP affects sensitivity of AT1R. Therefore, it seems that CDDP-induced nephrotoxicity is gender-dependent [9], and this difference might be dependent on RAS receptors. Notably, the AT2R/AT1R ratio is higher in females than males [9], and AT1R blockade may potentiate the role of AT2R. Consequently, female kidneys may receive a greater blood flow, leading to increased CDDP transport and subsequently more tissue damage (Table 5) [9].

## 5. Conclusion

RAS inhibitors by competitive inhibition of OATP1B1 or 1B3 increase CDDP-in contact with its transporter to the kidneys, and Ang II is enough effective in stimulating and increasing the activity of the OCT2, and both eventually increase kidney damage. Also, the renal vasodepressor arm of RAS, despite its effective role in improving some kidney diseases, worsens CDDP-induced nephrotoxicity in the female sex, which is related to the gender difference in kidney functions and responses in the presence of CDDP, as well as the effective performance of the depressor arm of RAS to increase RBF and ultimately transfer more CDDP to the kidneys. Moreover, inhibition of the pressor arm of RAS in males despite its effective role in RBF increasing somewhat reduces the renal damage caused by CDDP (affecting the KKS and antioxidant properties), which wholly expresses the different functions of kidneys in two sexes in the presence of physiological and pathological interventions of both RAS and CDDP. However, more studies are needed to make a precise decision about the role of the RAS in CDDP-induced renal injury.

## Figures and Tables

**Table 1:** Search syntax.

Databases	Search syntax	*N*
PubMed	((“Renin angiotensin system” OR “angiotensin II (1–7)” OR “angiotensin 1–7” OR “ACE2 Enzyme” OR “Mas receptor”) AND (Cisplatin) AND (Nephrotoxicity[tiab]))	15
Scopus	((ALL (“Renin angiotensin system”) OR ALL (“angiotensin II (1–7)”) OR ALL (“angiotensin 1–7”) OR ALL (“ACE2 Enzyme”) OR ALL(“mas-receptor”)) AND (ALL(“Cisplatin”)) AND (TITLE-ABS (“Nephrotoxicity”)))	158
ISI	((TS=(“Renin angiotensin system”) OR TS=(“angiotensin II (1–7)”) OR TS=(“angiotensin 1–7”) OR TS=(“ACE2 Enzyme”) OR TS=(“mas-receptor”)) AND (TS=(“Cisplatin”)) AND (TS=(“Nephrotoxicity”)))	21

**Table 2:** Characteristics of the studies conducted on investigation of the renin-angiotensin system in cisplatin-induced nephrotoxicity.

	Year	Author	Model	Cisplatin dose and duration	RAS components studied	Investigation	Results
1	2022	Kantauskaite et al. [14]	CDDP-induced nephrotoxicity	(MDCK cells were incubated for 4 h at 37°C with) 100 *µ*M	AT1R	The regulation of hOCT2 by AngII and its role in the development of CDDP toxicity	Reducing hOCT2 activity, by the inhibition of the AngII signaling pathway, protecting against CDDP nephrotoxicity
2	2022	Tsuji et al. [1]	CDDP	7 mg/kg (four times: on days 0, 7, 14, and 21)	RAS inhibitor (enalapril, losartan, and telmisartan)	The developing chronic kidney disease following repeated concomitant usage of CDDP and antihypertensive drugs	Exacerbating CDDP-induced nephrotoxicity via combinatorial treatment of CDDP and RAS inhibitors
3	2015	Nematbakhsh et al. [9]	CDDP-induced nephrotoxicity	2.5 mg/kg/day (continuous treatment: for a period of 1 week)	AT1R/AT2R	The role of CDDP in MAP response to graded Ang II infusion	Indicating the effect of CDDP on RAS and its related hemodynamic responses
4	2020	Estrela et al. [15]	CDDP-induced nephrotoxicity	20 mg/kg (single dose)	AT1R/AT2R/ACE	The preventive effect of enalapril on CDDP- induced nephrotoxicity	Restoring cell viability by cisplatin + enalapril treatment
5	2020	Kasaei et al. [16]	CDDP-induced nephrotoxicity	7.5 mg/kg (single dose)	AT1R and MasR	The effect of Ang1-7 and losartan on CDDP-induced nephrotoxicity	Promoting the CDDP-induced kidney damage via AT1R and MasR antagonists and Ang1-7 in female rats
6	2016	Nematbakhsh et al. [17]	CDDP-induced nephrotoxicity	2.5 mg/kg (continuous treatment: for 7 days)	ACE	The effect of an ACE inhibitor on CDDP-induced nephrotoxicity in male and female rats	Aggravating CDDP-induced nephrotoxicity by enalapril in female
7	2009	Saleh et al. [18]	CDDP-induced nephrotoxicity	7 mg/kg (single dose)/(renal cortical slices were incubated with) 2 mM	Losartan (Ang II receptor inhibitor)	The effects of losartan on CDDP-induced oxidative stress	Indicating protective effects of losartan against CDDP-induced nephrotoxicity
8	2012	Haghighi et al. [19]	CDDP-induced nephrotoxicity	7 mg/kg (single dose)	Losartan (Ang II receptor inhibitor)	The effects of losartan on CDDP-induced nephrotoxicity in male and female rats	Preventing CDDP-induced nephrotoxicity in male rats but promoting that in female rats by losartan
9	2012	Nematbakhsh et al. [20]	CDDP-induced nephrotoxicity	6 mg/kg (single dose, at day 3)	Losartan (Ang II receptor inhibitor)	Compare the effects of losartan and vitamin E as prophylaxes against CDDP-induced nephrotoxicity	Losartan as prophylaxes showed some nephroprotective effects. “Losartan and vitamin E” combination did not show a protective effect against CDDP-induced nephrotoxicity

hOCT2: Human organic cation transporter 2, CDDP: Cisplatin(Cis-diamminedichloroplatinum), MAP: Mean arterial pressure, MasR: Mas receptor, ACE: Angiotensin-converting enzyme, AT1R: Angiotensin II receptor type 1, AT2R: Angiotensin II type 2 receptor, RAS: Renin-angiotensin system, Ang II: Angiotensin II, Cr: Creatinine, BUN: Blood urea nitrogen, MasR: Mas receptor, MDCK cells: Madin–Darby canine kidney cells.

**Table 3:** Characteristics of studies indicating the therapeutic effects of renin-angiotensin system (RAS)-related interventions on cisplatin-induced nephrotoxicity.

	Year	Author	Model	Animal or human	RAS related drugs (dose)	Investigated receptor/enzyme/factor	Therapeutic effect(s)	(Probable) physiological route to the effects
4	2020	Estrela et al. [15]	CDDP-induced nephrotoxicity	Animal male C57Bl/6 mice	ACE inhibitor, enalapril (1.5 mg/kg)	AT1R/AT2R/ACE	Enalapril prevents CDDP nephrotoxicity	Enalapril inhibits the upregulation of B1R and carboxypeptidase M and the increased concentrations of kinin peptides through aminopeptidase activity restoration
7	2009	Saleh et al. [18]	CDDP-induced nephrotoxicity	Animal (in vivo) in vitro male albino rats	Losartan (Ang II receptor inhibitor) (60 mg/kg) Ang II receptor blocker	Ang II receptor	Losartan has protective effects against CDDP-induced nephrotoxicity and improves renal function	Losartan significantly restores normal serum Cr and BUN levels, and LDH leakage, and counteracts CDDP-induced lipid peroxidation and GSH attenuation
9	2012	Nematbakhsh et al. [20]	CDDP-induced nephrotoxicity	Animal	Losartan (Ang II receptor inhibitor) (10 mg/kg/day)	Ang II receptor	Losartan as prophylaxes shown some nephroprotective effects	Losartan decreased the kidney damage score compared to the CDDP group

B1R: Bradykinin receptor 1, Cr: Creatinine, LDH: Lactate dehydrogenase, Ang II: Angiotensin II, GSH: Glutathione, CDDP: Cisplatin, BUN: Blood urea nitrogen, ACE: Angiotensin-converting enzyme.

**Table 4:** Characteristics of studies indicating the pathologic effects of the renin-angiotensin system (RAS) on cisplatin-induced nephrotoxicity.

	Year	Author	Model	Animal or human	RAS related drugs (dose)	Investigated receptor/enzyme/factor	Pathological effect	(Probable) pathological route to the effects
1	2022	Kantauskaite et al. [14]	CDDP-induced nephrotoxicity	Cell line MDCK cells	Ang II	AT1R and hOCT2	Down-regulation of hOCT2 activity by the inhibition of AngII may protect against CDDP nephrotoxicity	Ang II stimulation of hOCT2 activity increases CDDP cellular toxicity and may involve in the development of CDDP nephrotoxicity

2	2022	Tsuji et al. [1]	CDDP-induced nephrotoxicity	Animal Male BALB/c mice	5 mg/kg, amlodipine/2.5 mg/kg, enalapril/10 mg/kg, telmisartan/10 mg/kg, losartan/5 mg/mL, AML	RAS inhibitors	Combinatorial treatment of CDDP and “enalapril or telmisartan” may exacerbate CDDP-induced nephrotoxicity and decrease renal function	1 Concomitant use of telmisartan increases BUN levels
								2 Enalapril or telmisartan may cause the competitive block of OATP1B1 or 1B3 in the liver, leading to increased uptake of CDDP by OCT2
								3 Enalapril or telmisartan may increase the *α*-SMA-stained area in this model

5	2020	Kasaei et al. [16]	CDDP-induced nephrotoxicity	Animal Female Wistar rats	Ang1-7 [30 *μ*g/kg/d (IP)] and Mas receptor (MasR) antagonist (A779, 100 *μ*g/kg/day IP)	AT1R and MasR	Cotreatment of CDDP with losartan, Ang1-7, or Ang1-7 plus A779 increases the serum BUN and Cr levels and kidney tissue damage score	Losartan and Ang1-7 may increase RBF which increases the amount of CDDP into the kidney and causes more kidney damage

6	2016	Zamani et al. [17]	CDDP-induced nephrotoxicity	Animal Male and female Wistar rats	Enalapril (30 mg/kg), IP as an ACE inhibitor	ACE	Enalapril aggravates CDDP-induced nephrotoxicity in females and decreases renal function	Enalapril intensified CDDP-induced serum BUN and Cr levels and kidney tissue damage in females

8	2012	Haghighi et al. [19]	CDDP-induced nephrotoxicity	Wistar rats	Losartan (Ang II receptor inhibitor) 10 mg/kg/day, IP	Ang II receptor	Losartan promotes CDDP-induced nephrotoxicity in females	Losartan increased CDDP-induced serum BUN and Cr levels and kidney tissue damage in females

IP injection: Intraperitoneal injection, MasR: Mas receptor, RBF: Renal blood flow, AngII: Angiotensin II, hOCT2: Human organic cation transporter 2, AT1R: Angiotensin II receptor type 1, CDDP: Cisplatin, MDCK cells: Madin–Darby canine kidney cells, RAS: Renin-angiotensin system, OATP1B1: Organic anion transporting polypeptides 1B1, Cr: Creatinine, BUN: Blood urea nitrogen, *α*-SMA: Alpha smooth muscle actin.

**Table 5:** Characteristics of studies indicating the pathologic effects of cisplatin on the renin-angiotensin system (RAS) in the nephrotoxicity model.

	Year	Author	Model	Animal or human	Investigated receptor/enzyme/factor	Effect of cisplatin on RAS	(Probable) pathologic pathway
3	2015	Dehghani et al. [9]	CDDP-induced nephrotoxicity	Animal male and female rats	Ang II/RAS receptors	CDDP affects sensitivity of the receptor to Ang II	CDDP promotes the vasoconstriction effect of Ang II in males more than that in females. The serum level of nitrite in male rats increased significantly via CDDP

RAS: Renin-angiotensin system, CDDP: Cisplatin, Ang II: Angiotensin II.

## Data Availability

The data supporting this systematic review are from previously reported studies and datasets, which have been cited.

## References

[1] Tsuji T., Hosoda A., Toriyama Y., Yoshida Y., Kohno T. (2022). Renin-angiotensin system inhibitors combined with cisplatin exacerbate cisplatin-induced nephrotoxicity in mice. *Translational Oncology*.

[2] Deegan P. M., Nolan C., Ryan M. P., Basinger M. A., Jones M. M., Hande K. R. (1995). The role of the renin-angiotensin system in cisplatin nephrotoxicity. *Renal Failure*.

[3] Ghaneialvar H., Kaffashian M. R., Salimi A. H. (2022). Protective effects of pretreatment or concomitant treatment with Hypericum extract on renal function and renal toxicity in cisplatin-induced nephrotoxicity. *Journal of Renal Injury Prevention*.

[4] dos Santos N. A. G., Carvalho Rodrigues M. A., Martins N. M., Dos Santos A. C. (2012). Cisplatin-induced nephrotoxicity and targets of nephroprotection: an update. *Archives of Toxicology*.

[5] Ozkok A., Edelstein C. L. (2014). Pathophysiology of cisplatin-induced acute kidney injury. *BioMed Research International*.

[6] Mehanna H., Robinson M., Hartley A. (2019). Radiotherapy plus cisplatin or cetuximab in low-risk human papillomavirus-positive oropharyngeal cancer (De-ESCALaTE HPV): an open-label randomised controlled phase 3 trial. *The Lancet*.

[7] Arora P., Rajagopalam S., Ranjan R. (2008). Preoperative use of angiotensin-converting enzyme inhibitors/angiotensin receptor blockers is associated with increased risk for acute kidney injury after cardiovascular surgery. *Clinical Journal of the American Society of Nephrology*.

[8] Ono M., Sakao Y., Tsuji T. (2015). Role of intrarenal (pro) renin receptor in ischemic acute kidney injury in rats. *Clinical and Experimental Nephrology*.

[9] Nematbakhsh M., Dehghani A., Saberi S. (2016). Cisplatin-induced nephrotoxicity alters blood pressure response to angiotensin II administration in rats. *Advanced Biomedical Research*.

[10] Maleki M., Aliboroni A., Kheiri A., Kaffashian M. R., Kheiry M. (2023). Association of the ACE2-Angiotensin1-7–Mas axis with lung damage caused by cigarette smoke exposure: a systematic review. *Reviews on Environmental Health*.

[11] Maleki M., Nematbakhsh M. (2016). Renal blood flow response to angiotensin 1-7 versus hypertonic sodium chloride 7.5% administration after acute hemorrhagic shock in rats. *International journal of vascular medicine*.

[12] Nematbakhsh M., Maleki M. (2019). Mas receptor antagonist (A799) alters the renal hemodynamics responses to angiotensin II administration after renal moderate ischemia/reperfusion in rats: gender related differences. *Research in Pharmaceutical Sciences*.

[13] Maleki M., Noorimotlagh Z., Mirzaee S. A. (2022). An updated systematic review on the maternal exposure to environmental pesticides and involved mechanisms of autism spectrum disorder (ASD) progression risk in children. *Reviews on Environmental Health*.

[14] Kantauskaite M., Hucke A., Snieder B., Ciarimboli G. (2022). Exacerbation of cisplatin cellular toxicity by regulation of the human organic cation transporter 2 through angiotensin II. *International Journal of Molecular Sciences*.

[15] Estrela G. R., Wasinski F., Gregnani M. F. (2020). Angiotensin-converting enzyme inhibitor protects against cisplatin nephrotoxicity by modulating kinin B1 receptor expression and aminopeptidase P activity in mice. *Frontiers in Molecular Biosciences*.

[16] Kasaei S., Pezeshki Z., Karimi F. (2021). Angiotensin 1-7 and losartan worsen the cisplatin induced nephrotoxicity in female rats. *Journal of Nephropharmacology*.

[17] Nematbakhsh M., Zamani Z., Eshraghi-Jazi F. (2016). Effect of enalapril in cisplatin-induced nephrotoxicity in rats; gender-related difference. *Advanced Biomedical Research*.

[18] Saleh S., Ain-Shoka A. A., El-Demerdash E., Khalef M. M. (2009). Protective effects of the angiotensin II receptor blocker losartan on cisplatin-induced kidney injury. *Chemotherapy*.

[19] Haghighi M., Nematbakhsh M., Talebi A. (2012). The role of angiotensin II receptor 1 (AT1) blockade in cisplatin-induced nephrotoxicity in rats: gender-related differences. *Renal Failure*.

[20] Nematbakhsh M., Ashrafi F., Safari T. (2012). Administration of vitamin E and losartan as prophylaxes in cisplatin-induced nephrotoxicity model in rats. *Journal of Nephrology*.

[21] Tian L., Liu H., Xie S. (2011). Effect of organic anion-transporting polypeptide 1B1 (OATP1B1) polymorphism on the single-and multiple-dose pharmacokinetics of enalapril in healthy Chinese adult men. *Clinical Therapeutics*.

[22] Lancaster C. S., Sprowl J. A., Walker A. L., Hu S., Gibson A. A., Sparreboom A. (2013). Modulation of OATP1B-type transporter function alters cellular uptake and disposition of platinum chemotherapeutics. *Molecular Cancer Therapeutics*.

[23] Hye Khan M. A., Abdul Sattar M., Abdullah N. A., Johns E. J. (2007). Cisplatin-induced nephrotoxicity causes altered renal hemodynamics in Wistar Kyoto and spontaneously hypertensive rats: role of augmented renal alpha-adrenergic responsiveness. *Experimental and Toxicologic Pathology*.

